# Associations between congenital cryptorchidism in newborn boys and levels of dioxins and PCBs in placenta

**DOI:** 10.1111/j.1365-2605.2011.01233.x

**Published:** 2012-06

**Authors:** H E Virtanen, J J Koskenniemi, E Sundqvist, K M Main, H Kiviranta, J T Tuomisto, J Tuomisto, M Viluksela, T Vartiainen, N E Skakkebaek, J Toppari

**Affiliations:** * Departments of Physiology and Paediatrics, University of TurkuTurku, Finland; † University Department of Growth and ReproductionRigshospitalet, Copenhagen, Denmark; ‡ Department of Environmental Health, National Institute for Health and WelfareKuopio, Finland

**Keywords:** cryptorchidism, dioxins, PCBs, placenta, testis

## Abstract

In animal studies, exposure to dioxins has been associated with disrupted development of the male reproductive system, including testicular maldescent. Some polychlorinated biphenyls (PCBs) have also dioxin-like effects. In addition, one previous case–control study has reported an association between congenital cryptorchidism and colostrum PCB levels. We performed a case–control study to evaluate whether congenital cryptorchidism in boys was associated with increased levels of dioxins or PCBs in placenta reflecting foetal exposure. In addition, associations between placenta levels of these chemicals and reproductive hormone levels in boys at 3 months were studied. Placentas were collected in a Danish–Finnish joint prospective cohort study on cryptorchidism (1997–2001). The boys were examined for cryptorchidism at birth and at 3 months. Altogether, 280 placentas [112 Finnish (56 cases, 56 controls) and 168 Danish (39 cases, 129 controls)] were analysed for 17 toxic polychlorinated dibenzo-*p*-dioxins and dibenzofurans (PCDD/Fs) and 37 PCBs (including 12 dioxin-like PCBs). Infant serum samples taken at 3 months were analysed for reproductive hormones. No significant differences between cases and controls were observed in either country in dioxin WHO-TEq levels (median 9.78 vs. 8.47 pg/g fat, respectively, in Finland, and 11.75 vs. 10.88 pg/g fat in Denmark) or PCB WHO-TEq levels (median 2.12 vs. 2.15 pg/g fat in Finland, 2.34 vs. 2.10 pg/g fat in Denmark) or total-TEq levels (median 11.66 vs. 10.58 pg/g fat in Finland, 13.94 vs. 13.00 pg/g fat in Denmark). Placenta WHO-TEq levels of dioxins were not associated with infant reproductive hormone levels at 3 months. In Finland, PCB WHO-TEq levels in placenta associated positively with infant LH levels. WHO-TEq levels of dioxins and PCBs and total-TEq levels were higher in Danish than Finnish samples. In conclusion, no association between placenta levels of dioxins or PCBs and congenital cryptorchidism was found. Significant country differences in chemical levels were observed.

## Introduction

Cryptorchidism, i.e. undescended testis, is one of the most common genital abnormalities in newborn boys. During the last two decades, the reported incidence of cryptorchidism in newborn boys with a birth weight ≥2500 g has varied between 2.1 and 8.4% in prospective studies using similar and clearly defined criteria (Virtanen & Toppari, 2008). Furthermore, some studies (Group JRHCS, 1992; Boisen *et al.*, 2004; Acerini *et al.*, 2009) have suggested an increasing trend in the incidence of congenital cryptorchidism when compared with previous studies (Buemann *et al.*, 1961; Scorer, 1964; Group JRHCS, 1992). Environmental chemicals with endocrine disrupting activities have been hypothesized to play a role in the increasing incidence of problems of male reproductive health (Sharpe & Skakkebaek, 1993; Toppari *et al.*, 1996; Sharpe, 2003).

Dioxins [i.e. polychlorinated dibenzo-*p*-dioxins (PCDDs) and related halogenated aromatic hydrocarbons, e.g. dibenzofurans (PCDFs)] and polychlorinated biphenyls (PCBs) are persistent environmental chemicals, which tend to accumulate in tissue lipid and in the food chain (Laisi *et al.*, 2008; Linden *et al.*, 2010). Dioxins are formed unintentionally, e.g. during unfavourable combustion processes such as waste incineration and during metal smelting and refining. Also, many chlorinated chemicals such as PCBs contain dioxins as impurities. Some PCBs have also dioxin-like effects (Linden *et al.*, 2010). PCBs have been used as insulators, fire retardants, lubricants for machinery and dielectric fluids in transformers (Guo *et al.*, 2004). Food of animal origin (dairy products, meat and fish) is the major exposure source of dioxins and PCBs for humans (Liem *et al.*, 2000). In animal experiments, various species, strain and dose dependent effects of dioxins have been observed (Birnbaum & Tuomisto, 2000). The developing immune, nervous and reproductive systems seem to be most sensitive to the adverse effects of dioxins.

In rats and pigs, *in utero* and lactational exposure to 2,3,7,8-TCDD (the most toxic dioxin congener) has been associated with disrupted descent of the testes (Mably *et al.*, 1992; Barthold *et al.*, 1999). In one previous case–control study, the risk of congenital cryptorchidism was increased in boys who had the highest exposure to seven non-planar PCBs in maternal colostrum (Brucker-Davis *et al.*, 2008). The aim of this study was to evaluate whether congenital cryptorchidism in man was associated with contemporary dioxin and PCB levels in placenta. In addition, we studied associations between the levels of dioxins and PCBs in placenta and reproductive hormone levels in boys at 3 months of age.

## Subjects and methods

The study was a nested case–control study within a prospective cohort. The study included maternal placenta samples representing cryptorchid or healthy boys who had participated in the Danish–Finnish joint prospective cohort study on the incidence and risk factors of congenital cryptorchidism and hypospadias (Virtanen *et al.*, 2001; Boisen *et al.*, 2004, 2005). The boys were born in 1997–2001 at the Turku University Hospital or at the University Hospital of Copenhagen (Rigshospitalet and Hvidovre Hospital). They were examined for cryptorchidism at birth (preterm cases at the expected date of delivery) and at 3 months (preterm cases 3 months after the expected date). In preterm boys with undescended testis, cryptorchidism was diagnosed only if the testis remained undescended at the expected date of delivery. The standardized recruitment criteria and examination technique, and the observed prevalence rates of cryptorchidism (Boisen *et al.*, 2004) and some results on chemical levels in breast milk and placenta samples have been published previously (Shen *et al.*, 2005, 2006, 2007, 2008; Damgaard *et al.*, 2006; Main *et al.*, 2006b, 2007; Krysiak-Baltyn *et al.*, 2010). Boys with normally descended testes (including retractile testes) were classified as controls in this study. Boys with at least one undescended testis located high in the scrotum or in a higher position (Boisen *et al.*, 2004) were classified as cryptorchid cases.

The study was performed according to the Helsinki II declaration, and was approved by the local Finnish and Danish ethics committees and the Danish Data Protection Agency. Oral and written informed consent was given by the parents.

Placenta dioxin and PCB levels were used as a proxy for foetal exposure, as placenta levels have been shown to correlate with levels in third trimester maternal serum and umbilical cord serum (Wang *et al.*, 2004). Midwives collected and froze (−20 °C) the whole placentas immediately after birth in double polyethylene bags. In Denmark, placentas were collected from all participants. In Finland, as a result of limited storage space, biological samples were collected from cases and matched controls [matching criteria were date of birth (±2 weeks), parity, gestational age (±1 week), smoking during pregnancy (yes/no) and maternal diabetes (yes/no)]. Information on smoking was based on a prenatal questionnaire and telephone interview (the latter only in Denmark). As a result of limited funding, only part of the collected samples (280 placentas) was selected for prospectively planned exposure measurements. In Denmark, all available case placentas were selected, and control placentas were selected randomly from the biobank. In Finland, 56 matched case–control placenta pairs were selected for the exposure measurements.

Altogether 112 Finnish (56 cases, 56 controls) and 168 Danish (39 cases, 129 controls) placentas were analysed for 17 toxic PCDD/Fs and 37 PCBs. The concentrations of dioxins and PCBs in placenta are presented as sums of 17 or 37 different congener concentrations, respectively, and as WHO-TEq values, i.e. WHO-recommended 2,3,7,8-TCDD equivalents for the 17 PCDD/Fs or for the 12 dioxin-like PCBs (non-*ortho*-PCBs 77, 81, 126, 169 and mono-*ortho*-PCBs 105, 114, 118, 123, 156, 157, 167 and 189). In addition, a total-TEq value is presented (sum of the WHO-TEqs for dioxins and dioxin-like PCBs). WHO-TEq value is calculated by multiplying the concentration of each congener by a toxic equivalency factor (TEF), and summing up all equivalents (Van den Berg *et al.*, 1998, 2006). As the samples were collected in 1997–2001, WHO 1997 TEF values (Van den Berg *et al.*, 1998) were used in this study to allow comparison with other studies from close time periods.

During the examination at the age of 3 months, a 4 mL venous non-fasting blood sample was collected from the boys. This sample represents the time of minipuberty, i.e. the time of short physiological activation of the hypothalamic-pituitary-testicular axis during the first few months of life (Andersson *et al.*, 1998). The success rate of obtaining a blood sample was 69% in the case-control study. The blood samples were centrifuged after clotting, and sera were separated and stored at −20 °C until analysed. All samples were analysed as duplicates at Rigshospitalet and the technicians were blinded as to the sample origin. To minimize any effect of interassay variation, samples from both countries and from both cases and controls were included in each run.

Time-resolved immunofluorometric assays (Delfia, Wallac Inc., Turku, Finland) were used for serum follicle-stimulating hormone (FSH), luteinizing hormone (LH) and sex hormone-binding globulin (SHBG). The detection limits were 0.06 IU/L, 0.05 IU/L and 0.23 nmol/L, respectively. The intra- and interassay coefficients of variation (CVs) were <5% in the gonadotropin assays and <6% for SHBG. Radioimmunoassay (Coat-a-Count; Diagnostic Products Corp., Los Angeles, CA, USA) was used in testosterone measurements, with detection limit 0.23 nmol/L and intra- and interassay CVs <10%. Free testosterone index was calculated (testosterone × 100)/SHBG. A double antibody enzyme-immunometric assay (Main *et al.*, 2006a) was used for inhibin B. The detection limit was 20 pg/mL and intra- and interassay CVs were <15 and <18%, respectively. Ratios between hormones were calculated: LH/testosterone, LH/free testosterone, FSH/inhibin B. The number of sex hormone analyses per boy depended on sample volume.

All PCDD/F and PCB analyses were performed at the Department of Environmental Health in Kuopio, Finland. Placentas were defrosted; the umbilical cord and all readily removable membranes were discarded. Whole placentas were homogenized in a mixer (Büchi Mixer B-400; Büchi Laboratories AG, Flawil, Switzerland) and 75 g of the homogenate was lyophilized. Dried homogenate was pulverized in a mortar, and slurry was made by adding dichloromethane and cyclohexane (1 : 1 vol/vol) and concentrated sulphuric acid. This slurry was spiked with sixteen ^13^C-labelled 2,3,7,8-chlorine substituted PCDD/F internal standards, with four ^13^C-labelled non-*ortho*-PCBs standards (PCB 77, 81, 126 and 169) and thirteen other ^13^C-labelled PCB standards [PCB 30 (^12^C-labelled), 52, 80, 101, 105, 118, 138, 153, 156, 157, 170, 180, 194 and 209] (Wellington Laboratories Inc., Guelph, ON, Canada). Fat content was determined at the Institute of Ecological Chemistry in Neuherberg, Germany (Shen *et al.*, 2005, 2006, 2007, 2008).

The procedure for fat removal and sample clean up has been described previously (Kiviranta *et al.*, 2004). The quantification of 17 PCDD/F congeners (all 2,3,7,8-chlorine substituted toxic PCDD/F congeners) and of 37 PCB congeners [four non-*ortho* (77, 81, 126 and 169), eight mono-*ortho* (PCB 105, 114, 118, 123, 156, 157, 167 and 189) and 25 other PCB congeners (18, 28/31, 33, 47, 49, 51, 52, 60, 66, 74, 99, 101, 110, 122, 128, 138, 141, 153, 170, 180, 183, 187, 194, 206 and 209)] was performed by selective-ion recording using a high resolution mass spectrometer, VG 70-250 SE (VG Analytical, UK) at a resolution of 10 000. Gas chromatographic separation of the PCDD/Fs and PCBs was performed using a HP 6890 gas chromatograph with fused silica capillary column (DB-DIOXIN (60 m, 0.25 mm, 0.15 μm); J&W Scientific, Folsom, CA, USA). As recovery standards for internal PCDD/F standards two ^13^C-labelled PCDD congeners were used, namely ^13^C 1,2,3,4-TCDD and ^13^C 1,2,3,7,8,9-HxCDD. As a recovery standard for internal non-*ortho*-PCB standards, ^13^C- PCB-60 was used, and for internal mono- and di-*ortho*-PCB standards, ^12^C-PCB-159 was used.

Both the technicians and chemists were blinded to the case/control status of the samples. Laboratory and cross-sample contamination was monitored by analysing procedural blank samples. The concentrations of blanks were much lower than the concentrations in placenta, on average 1.1 and 4.1% of the WHO-TEq for dioxins and PCBs respectively.

Recoveries of individual internal PCDD/F and PCB standards were >60%. Median limit of quantification (LOQ) of a single congener for placentas corresponding to a signal to noise ratio of 3:1, was 0.32 pg/g fat (range: 0.12–0.25 pg/g fat for tetra to hepta chlorinated and 1.2 pg/g fat for octa chlorinated PCDD/F congeners), for non-*ortho*-PCBs 0.17 pg/g fat and 0.01 ng/g fat for mono- and di-*ortho*-PCBs. In the in-house placenta control samples, the CV for dioxin WHO-TEq was 5.6%, and for PCB WHO-TEq, the CV was 13%. Concentrations < LOQ were considered to be equal to nil (lower bound results) in accordance with other studies (Chao *et al.*, 2007; Feshin *et al.*, 2008). During the study period, the laboratory successfully participated in interlaboratory comparison studies of PCDD/Fs and PCBs in different biological matrices (Becher *et al.*, 2001; Småstuen Haug & Becher, 2004, 2005). The Finnish Accreditation Service, FINAS, has certified the laboratory (T077) in performing PCDD/F and PCB analyses in biological samples according to the EN ISO/IEC 17025 standard.

## Statistics

Wilcoxon two-sample test, Chi-square test and Fisher’s exact test were used when comparing population characteristics between cases and controls in each country. As the Finnish samples were matched for gestational age, parity, maternal smoking and diabetes, case–control differences in these factors were not tested. Logarithmic and square root transformations were used when necessary to approximate normal distributions of chemical and hormonal data.

Logistic regression analysis was used when evaluating case–control differences in Danish placentas. Maternal age, parity, body mass index (BMI) and date of childbirth were included in the original models, as the Danish samples were not matched, and these factors could possibly affect the chemical levels. In addition, the original models included well-described risk factors of cryptorchidism, i.e. prematurity and weight for gestational age (small for gestational age/appropriate for gestational age/large for gestational age) and gestational age. The final model containing only significant confounders was achieved by backward selection. As the Finnish placenta material consisted of matched pairs, case–control differences in placentas were evaluated using conditional logistic regression analysis including maternal age and BMI, as these could affect the chemical levels.

Regression analysis (without and with adjustment for confounding factors) was used when evaluating differences in exposure between countries. This analysis included only control samples. The original adjusted model included maternal age, maternal smoking and diabetes during pregnancy, BMI, parity and date of delivery within the cohort and gestational age. Only significant variables (date of delivery within the cohort, maternal age, parity, BMI and/or smoking) were left in the final models.

When evaluating associations between serum hormone levels and PCDD/F WHO-TEq levels and PCB WHO-TEq levels in placenta, regression analysis was used, and the model included the exact age at infant blood sampling and status of the child (cryptorchid case/control). Hormonal associations were analysed separately in each country. The results are presented if both the overall model and the main effect of dioxin/PCB level were significant. *p*-values below 0.05 were considered as statistically significant. The statistical analyses were performed using SAS for Windows, release 9.1.

## Results

### Study population

There were no significant differences between cases and controls in maternal age, BMI or weight for gestational age in either country (Table 1). In the Finnish group, maternal diabetes during pregnancy was significantly more frequent among cases, *p* = 0.001, as it was not always possible to find a control fulfilling this matching criterion. In the Danish material, there were no significant differences between the cases and controls in maternal parity, diabetes or smoking during pregnancy. In Danish boys, gestational age was significantly lower in cryptorchid boys than in controls, *p* < 0.001, and Danish cases had significantly lower birth weight than the controls, *p* = 0.04. Spontaneous testicular descent by the age of 3 months was significantly more frequent in Danish than in Finnish cases, *p* < 0.001.

**Table 1:** Description of study population representing the placenta samples. Median (range) or numbers are given

	Finland			Denmark		
		
	control (*n *= 56)	case (*n *= 56)	*p*-value	control (*n *= 129)	case (*n *= 39)	*p*-value
Maternal age	28.1(19.9–38.5)	29.1 (19.2–42.3)	0.35	31.0 (19.8–42.5)	29.5 (25.7–45.7)	0.72
BMI (kg/m^2^)	22.3 (17.4–32.1)	23.1 (17.7–38.5)	0.07	22.1 (17.4–37.6)	21.5 (17.8–36.1)	0.20
smoking (yes/no)	7/49	10/46	NA	41/87	11/28	0.65
Diabetes (yes/no)	0/55	10/46	NA	2/127	0/39	1.00
Parity						
1	31	31	NA	81	26	0.90
2	19	19		36	10	
≥3	6	6		12	3	
Gestational age (days)	280 (255–294)	279 (256–297)	NA	283 (252–299)	276 (195–294)	<0.001
Weight for gest. age (%)	−0.74 (−21.9–29.2)	−1.12 (−34.5–27.3)	0.91	0.29 (−24.7–51.7)	−0.83 (−39.5–44.4)	0.92
Birthweight (kg)	3.54 (2.84–4.73)	3.63 (2.51–4.66)	0.81	3.63 (2.29–5.68)	3.45 (0.75–4.75)	0.04
Cryptorchid at 3 months	0	33		0	8	
Premature	3	1		6	6	
Born as SGA	0	3		5	2	
Infant blood sample	44	35		88	25	

BMI, body mass index; NA, not applicable; SGA, small for gestational age.

### Case–control differences

**Figure 1:**
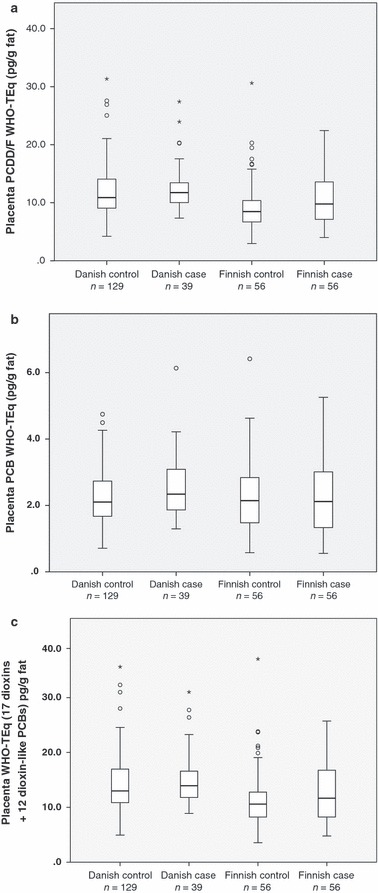
(a) PCDD/F WHO-TEq levels in placenta in Danish and Finnish controls and cases. Median, lower and upper quartile and range are given. (Outliers marked with stars and circles). (b) PCB WHO-TEq levels in placenta in Danish and Finnish controls and cases. Median, lower and upper quartile and range are given. (Outliers marked with circles). (c) Total WHO-TEq levels in placenta in Danish and Finnish controls and cases. Median, lower and upper quartile and range are given. (Outliers marked with stars and circles).

There were no significant differences between cases and controls in the sum of 17 dioxins or in the dioxin WHO-TEq levels either in Finland or in Denmark (Fig. 1a, Table 2). When including the analysis only the six dioxin congeners quantifiable in all placentas (2,3,7,8-TCDD, 2,3,4,7,8-PeCDF, 1,2,3,7,8-PeCDD, 1,2,3,6,7,8-HxCDD, 1,2,3,4,6,7,8-HpCDD and OCDD), no significant differences between cases and controls for individual congeners, their sum or the respective dioxin WHO-TEq levels were observed in either country.

**Table 2:** Placenta dioxin levels (pg/g fat) (lower bound results, i.e. concentrations below limit of quantification = 0). Percentage of quantifiable samples and median (range) are given

	%	FIN controls (*n *= 56)	%	FIN cases (*n *= 56)	Case vs. control *p*-value^a^	%	DK controls (*n *= 129)	%	DK cases (*n *= 39)	Case vs. control *p*-value^b^
2,3,7,8-TCDF	91	0.27 (0–0.98)	95	0.36 (0–0.96)	0.03	90	0.25 (0–1.12)	85	0.21 (0–0.92)	0.35
2,3,7,8-TCDD	100	0.84 (0.34–3.09)	100	0.95 (0.31–1.99)	0.91	100	1.19 (0.50–2.82)	100	1.31 (0.79–3.76)	0.13
1,2,3,7,8-PeCDF	89	0.21 (0–1.33)	89	0.26 (0–0.81)	0.60	74	0.20 (0–0.99)	51	0.12 (0–0.70)	0.02
2,3,4,7,8-PeCDF	100	6.15 (1.94–31.74)	100	7.92 (2.81–21.62)	0.22	100	7.07 (2.81–19.01)	100	6.91 (4.03–15.93)	0.76
1,2,3,7,8-PeCDD	100	2.88 (1.05–8.94)	100	3.25 (1.23–7.11)	0.53	100	4.49 (1.69–16.38)	100	5.12 (2.68–13.15)	0.57
1,2,3,4,7,8-HxCDF	100	1.58 (0.64–3.24)	100	1.68 (0.50–3.22)	0.67	99	2.06 (0–3.88)	100	1.93 (1.00–3.76)	0.23
1,2,3,6,7,8-HxCDF	98	0.78 (0–1.93)	98	1.01 (0–1.79)	0.95	98	1.15 (0–2.56)	100	1.11 (0.54–2.28)	0.59
2,3,4,6,7,8-HxCDF	50	0.09 (0–0.96)	63	0.28 (0–0.78)	0.47	72	0.37 (0–1.45)	67	0.37 (0–1.29)	0.74
1,2,3,7,8,9-HxCDF	2	0 (0–0.45)	0	0 (0–0)	NA	0	0 (0–0)	0	0 (0–0)	NA
1,2,3,4,7,8-HxCDD	82	0.97 (0–2.86)	95	1.03 (0–2.48)	0.08	100	3.35 (0.83–11.85)	100	3.36 (1.46–7.47)	0.72
1,2,3,6,7,8-HxCDD	100	7.25 (2.60–14.45)	100	7.75 (1.86–14.93)	0.85	100	5.98 (1.69–17.91)	100	6.17 (3.84–14.22)	0.88
1,2,3,7,8,9-HxCDD	96	1.94 (0–8.85)	100	2.19 (0.84–8.73)	0.90	100	2.33 (0.71–16.00)	100	2.60 (1.45–9.86)	0.73
1,2,3,4,6,7,8-HpCDF	100	1.80 (0.39–5.79)	98	1.74 (0–5.08)	0.21	95	1.54 (0–33.92)	92	1.03 (0–4.37)	0.01
1,2,3,4,7,8,9-HpCDF	4	0 (0–0.15)	5	0 (0–0.25)	NA	2	0 (0–0.50)	0	0 (0–0)	NA
1,2,3,4,6,7,8-HpCDD	100	11.79 (3.75–40.22)	100	10.69 (4.70–45.44)	0.52	100	9.18 (2.74–33.90)	100	9.13 (2.16–36.48)	0.32
OCDF	4	0 (0–1.53)	0	0 (0–0)	NA	5	0 (0–29.79)	0	0 (0–0)	NA
OCDD	100	82.15 (26.87–314.05)	100	69.74 (32.63–254.95)	0.17	100	76.59 (23.37–255.87)	100	70.64 (29.23–302.18)	0.32
Sum of 17 congeners		118.14 (40.40–384.50)		114.30 (55.22–361.36)	0.31		123.73 (45.16–325.10)		113.32 (50.64–367.53)	0.31
WHO-TEq		8.47 (2.97–30.62)		9.78 (4.02–22.43)	0.39		10.88 (4.22–31.35)		11.75 (7.35–27.42)	0.71
Sum of 6 congeners^c^		110.43 (36.91–376.33)		104.16 (50.72–343.02)	0.29		110.07 (40.28–311.31)		103.73 (44.96–354.79)	0.38
WHO-TEq of 6 cong.^c^		7.70 (2.72–29.34)		9.01 (3.75–21.00)	0.39		9.98 (3.82–28.03)		10.71 (6.86–25.93)	0.67

NA, not applicable; BMI, body mass index.

a Mother’s age and BMI included in the model.

b Gestational age included in the model.

c Congeners measurable in all placenta samples.

There were no significant case–control differences in the sum of 37 PCBs or in the PCB WHO-TEq levels in either country (Fig. 1b and Table 3). When including only the 21 PCB congeners quantifiable in all placenta samples (PCB 28/31, 60, 66, 74, 99, 105, 114, 118, 126, 138, 153, 156, 157, 167, 169, 170, 180, 183, 187, 189 and 194) in the analysis, no significant differences between cases and controls in their sum or in the respective PCB WHO-TEq levels were observed in Finland or in Denmark. When evaluating the levels of the above mentioned 21 single PCB congeners quantifiable in all placenta samples, Danish cases had higher levels of PCB 126 than Danish controls, but there were no other significant case–control differences in either country.

**Table 3:** Placenta PCB levels (lower bound results, i.e. concentrations below limit of quantification = 0). Percentage of quantifiable samples and median (range) are given

	%	Controls FI (*n *= 56)	%	Cases FI (*n *= 56)	Case vs. control *p*-value^a^	%	Controls DK (*n *= 129)	%	Cases DK (*n *= 39)	Case vs. control *p*-value^b^
PCB 18 (ng/g fat)	75	0.03 (0–0.18)	91	0.06 (0–0.12)	0.01	99	0.07 (0–0.91)	100	0.06 (0.02–0.20)	0.44
PCB 28/31 (ng/g fat)	100	0.47 (0.16–1.89)	100	0.48 (0.25–3.49)	0.33	100	0.47 (0.19–3.59)	100	0.57 (0.21–1.06)	0.32
PCB 33 (ng/g fat)	77	0.04 (0–0.16)	96	0.04 (0–0.12)	0.08	99	0.05 (0–0.40)	100	0.04 (0.02–0.09)	0.26
PCB 47 (ng/g fat)	100	0.05 (0.01–0.21)	100	0.06 (0.02–0.24)	0.02	98	0.06 (0–1.07)	100	0.07 (0.03–0.11)	0.80
PCB 49 (ng/g fat)	95	0.06 (0–0.46)	100	0.06 (0.02–0.25)	0.98	98	0.04 (0–0.25)	97	0.04 (0–0.09)	0.10
PCB 51 (ng/g fat)	20	0 (0–0.02)	20	0 (0–0.03)	0.51	4	0 (0–0.06)	13	0 (0–0.02)	0.18
PCB 52 (ng/g fat)	100	0.15 (0.03–2.51)	100	0.22 (0.03–1.19)	0.72	98	0.09 (0–1.88)	100	0.09 (0.03–0.13)	0.17
PCB 60 (ng/g fat)	100	0.09 (0.02–0.35)	100	0.10 (0.03–0.36)	0.87	100	0.10 (0.03–4.91)	100	0.10 (0.05–0.34)	0.74
PCB 66 (ng/g fat)	100	0.29 (0.07–1.78)	100	0.30 (0.08–1.02)	0.57	100	0.24 (0.07–6.34)	100	0.26 (0.12–0.82)	0.94
PCB 74 (ng/g fat)	100	0.95 (0.38–2.43)	100	0.99 (0.39–14.59)	0.21	100	1.05 (0.50–13.24)	100	1.12 (0.72–2.18)	0.78
PCB 77 (pg/g fat)	91	8.86 (0–36.13)	91	5.14 (0–29.30)	0.005	91	3.23 (0–23.90)	100	5.37 (0.43–13.69)	0.02
PCB 81 (pg/g fat)	73	0.48 (0–1.21)	89	0.60 (0–1.85)	0.03	91	0.52 (0–2.05)	95	0.50 (0–1.27)	0.51
PCB 99 (ng/g fat)	100	1.23 (0.37–3.16)	100	1.19 (0.42–2.64)	0.91	100	1.25 (0.47–3.24)	100	1.29 (0.79–1.94)	0.58
PCB 101 (ng/g fat)	100	0.35 (0.04–7.64)	98	0.27 (0–3.74)	0.12	71	0.06 (0–0.64)	87	0.10 (0–0.33)	0.16
PCB 105 (ng/g fat)	100	0.53 (0.14–3.44)	100	0.70 (0.10–2.09)	0.44	100	0.43 (0.15–3.03)	100	0.45 (0.24–0.97)	0.63
PCB 110 (ng/g fat)	93	0.28 (0–19.32)	100	0.21 (0.02–5.25)	0.15	65	0.03 (0–0.90)	90	0.08 (0–0.16)	0.02
PCB 114 (ng/g fat)	100	0.08 (0.02–0.20)	100	0.10 (0.03–0.38)	0.32	100	0.11 (0.04–0.34)	100	0.11 (0.06–0.37)	0.86
PCB 118 (ng/g fat)	100	2.02 (0.73–8.67)	100	2.62 (0.61–6.64)	0.62	100	2.05 (0.88–5.96)	100	2.13 (1.23–4.69)	0.37
PCB 122 (ng/g fat)	27	0 (0–0.06)	27	0 (0–0.04)	0.19	0	0 (0–0)	0	0 (0–0)	NA
PCB 123 (ng/g fat)	96	0.09 (0–0.56)	98	0.12 (0–0.44)	0.50	100	0.07 (0.03–0.38)	97	0.08 (0–0.20)	0.70
PCB 126 (pg/g fat)	100	9.47 (1.93–38.39)	100	9.58 (1.22–27.01)	0.32	100	8.30 (2.58–21.16)	100	10.91 (4.85–21.26)	0.01
PCB 128 (ng/g fat)	100	0.17 (0.02–3.26)	100	0.20 (0.02–0.90)	0.12	99	0.12 (0–0.46)	100	0.14 (0.04–0.22)	0.66
PCB 138 (ng/g fat)	100	6.22 (1.80–18.87)	100	6.32 (2.34–18.36)	0.80	100	8.27 (2.34–19.94)	100	8.15 (3.68–16.11)	0.49
PCB 141 (ng/g fat)	98	0.10 (0–0.80)	95	0.09 (0–0.47)	0.04	50	0 (0–0.10)	90	0.04 (0–0.11)	<0.001
PCB 153 (ng/g fat)	100	9.35 (2.75–28.04)	100	9.96 (3.69–33.66)	0.82	100	14.15 (3.90–34.55)	100	14.10 (7.21–36.11)	0.76
PCB 156 (ng/g fat)	100	0.92 (0.22–2.22)	100	1.07 (0.25–4.74)	0.64	100	1.45 (0.37–3.89)	100	1.36 (0.62–5.73)	0.51
PCB 157 (ng/g fat)	100	0.15 (0.04–0.40)	100	0.16 (0.04–0.86)	0.59	100	0.22 (0.06–0.58)	100	0.21 (0.10–0.79)	0.67
PCB 167 (ng/g fat)	100	0.15 (0.03–0.63)	100	0.20 (0.05–0.55)	0.17	100	0.24 (0.08–0.62)	100	0.24 (0.10–0.92)	0.32
PCB 169 (pg/g fat)	100	3.91 (1.16–15.58)	100	4.52 (1.05–11.40)	0.92	100	6.95 (1.74–17.70)	100	6.64 (2.83–23.75)	0.60
PCB 170 (ng/g fat)	100	2.73 (0.76–8.54)	100	2.99 (0.86–14.90)	0.44	100	4.84 (1.01–12.78)	100	4.61 (2.19–19.85)	0.44
PCB 180 (ng/g fat)	100	5.56 (1.44–14.92)	100	5.88 (1.97–28.37)	0.52	100	9.65 (2.03–25.21)	100	8.88 (4.07–37.89)	0.47
PCB 183 (ng/g fat)	100	0.74 (0.22–2.25)	100	0.72 (0.33–2.57)	0.80	100	1.09 (0.27–3.13)	100	1.11 (0.48–2.24)	0.37
PCB 187 (ng/g fat)	100	1.52 (0.37–4.92)	100	1.57 (0.68–8.10)	0.63	100	2.07 (0.53–5.24)	100	2.08 (0.83–6.00)	0.91
PCB 189 (ng/g fat)	100	0.10 (0.02–0.36)	100	0.11 (0.03–0.51)	0.94	100	0.17 (0.03–0.52)	100	0.16 (0.07–0.73)	0.80
PCB 194 (ng/g fat)	100	0.67 (0.16–2.02)	100	0.71 (0.26–3.70)	0.78	100	1.16 (0.24–2.96)	100	1.01 (0.42–5.18)	0.27
PCB 206 (ng/g fat)	96	0.17 (0–0.52)	98	0.14 (0–0.70)	0.54	100	0.25 (0.02–0.66)	100	0.22 (0.10–0.70)	0.40
PCB 209 (ng/g fat)	98	0.09 (0–0.25)	100	0.10 (0.02–0.28)	0.48	100	0.25 (0.06–0.63)	100	0.22 (0.11–0.78)	0.22
Sum of all PCBs (ng/g fat)		38.93 (10.73–101.33)		42.14 (15.02–132.63)	0.54		52.26 (13.97–114.26)		47.75 (25.50–145.33)	0.58
PCB WHO-TEq (pg/g fat)		2.15 (0.57–6.42)		2.12 (0.56–5.26)	0.30		2.10 (0.71–4.75)		2.34 (1.29–6.14)	0.26
Sum of 21 PCBs^c^ (ng/g fat)		35.68 (10.43–98.61)		39.16 (13.79–126.28)	0.92		51.36 (13.60–111.93)		46.20 (24.69–142.80)	0.59
WHO-TEq of PCBs^c^ (pg/g fat)		2.14 (0.57–6.38)		2.10 (0.55–5.24)	0.31		2.10 (0.71–4.73)		2.33 (1.29–6.12)	0.26

NA, not applicable; PCB, polychlorinated biphenyl; BMI, body mass index.

a Mother’s age and BMI included in the model.

b Gestational age included in the model.

c Congeners measurable in all placenta samples.

No significant differences between controls and cases were observed in either country for total WHO-TEq levels, i.e. total-TEq levels representing both dioxins and dioxin-like PCBs (Fig. 1c). Median (range) placenta total-TEq levels were 10.58 (3.54–37.04) pg/g fat and 11.66 (4.78–25.73) pg/g fat, in Finnish controls and cases, respectively, adjusted *p* = 0.64. In the Danish material, the respective levels were 13.00 (4.93–35.61) pg/g fat vs. 13.94 (8.89–31.01) pg/g fat, adjusted *p* = 0.60.

### Hormonal associations

In the Finnish material, PCB WHO-TEq levels in placenta were positively associated with the infant serum levels of LH at the age of 3 months (*b* = 0.47, *p* = 0.01). No other statistically significant associations between PCDD/F or PCB WHO-TEq levels in placenta and reproductive hormone levels at 3 months were observed.

### Country differences

Only control samples were included when evaluating differences between the countries in the levels of dioxins and PCBs in placenta. There was no significant difference in the sum of dioxins. However, the PCDD/F WHO-TEq level was significantly higher in Denmark than in Finland (adjusted *p* < 0.001). The sum of PCBs was significantly higher in Danish samples than in Finnish samples (adjusted *p* < 0.001), and after adjustment for confounding factors, also the PCB WHO-TEq level was significantly higher in Danish samples than in Finnish samples (adjusted *p* = 0.03, significant difference only after adjustment). The total-TEq level was also significantly higher in Denmark than in Finland (*p* < 0.001). As a result of the observed differences between Denmark and Finland, countries were analysed separately when evaluating case–control differences in chemical levels or associations between hormone and chemical levels.

## Discussion

No association between exposure to current background levels of dioxins and PCBs (evaluated as placenta levels) and congenital cryptorchidism was found in our study. In a previous French study, Brucker-Davis *et al.* reported an association between PCB levels (sum of PCBs 28, 52, 101, 118, 138, 153 and 180) in colostrum and congenital cryptorchidism; the risk of cryptorchidism was increased in the highest exposure class to PCBs (Brucker-Davis *et al.*, 2008). Brucker-Davis *et al.* also evaluated the sum of the seven PCBs in cord blood in the same study and found no association with congenital cryptorchidism (Brucker-Davis *et al.*, 2008). Breast milk contains significantly higher fat levels than serum or placenta and persistent chemicals may be excreted and stored differently in these biological matrices. McGlynn *et al.* evaluated levels of 11 PCBs (PCBs 28, 52, 74, 105, 118, 138, 153, 170, 180, 194 and 203) in maternal third trimester serum from the 1960s, and found either no significant association with cryptorchidism in the sons (McGlynn *et al.*, 2009). In a German study, Hosie *et al.* evaluated the levels of six PCBs (28, 52, 101, 138, 153, 180) and their sum in fat biopsies representing boys operated for cryptorchidism (up to the age of 12 years) and boys operated for other reasons (Hosie *et al.*, 2000). No significant differences in the PCB levels between the two groups were found (Hosie *et al.*, 2000).

In the Danish–Finnish joint prospective cohort study on cryptorchidism, we also evaluated association between congenital cryptorchidism and the chemical pattern in breast milk (including polybrominated biphenyls (PBBs), polybrominated diphenyl ethers (PBDEs), phthatales, organochlorine pesticides, PCBs and dioxins) (K. Krysiak-Baltyn, J. Toppari, N.E. Skakkebaek, T.S. Jensen, H.E Virtanen, K-W. Schramm, H. Shen, T. Vartiainen, H. Kiviranta, O. Taboureau, K. Audouze, S. Brunak & K.M. Main, unpublished data). Previous studies on the association between congenital cryptorchidism in man and exposure to dioxins are scarce. A case–control study nested in a cohort of British Columbia sawmill workers suggested a possible positive association between paternal cumulative exposure to dioxin-contaminated chlorophenates during pregnancy and cryptorchidism in the son (Dimich-Ward *et al.*, 1996).

In chemical production plant workers exposed to dioxins 15–37 years earlier, serum TCDD concentration was positively and significantly related with LH and FSH levels and inversely related with total testosterone levels in linear regression analyses after adjustment for confounders (Egeland *et al.*, 1994). Similarly, in a study of young men from the general Flemish population, an increase in serum dioxin-like activity (CALUX-TEQs >16 pg/L) was associated with a decrease in total and free serum testosterone (Dhooge *et al.*, 2006). Also, in a prospective cohort study of U.S. Air Force veterans, serum testosterone levels were negatively associated with serum TCDD levels (Gupta *et al.*, 2006). In an Italian study, breast-fed sons of dioxin-exposed mothers (Seveso accident) had, in adulthood, poorer semen quality and higher FSH and lower inhibin B levels than breast-fed controls (Mocarelli *et al.*, 2011). Also, men exposed prenatally to PCBs and PCDFs in the Yu-Cheng accident in Taiwan were reported to have poorer semen quality than unexposed controls, but no data on hormone levels was given (Guo *et al.*, 2000).

In some animal studies both *in utero* and adult exposure to 2,3,7,8-TCDD has been associated with reduced foetal and adult testosterone levels respectively (Moore *et al.*, 1985; Mably *et al.*, 1992). However, results on the impact of TCDD exposure on testosterone levels are contradictory (Foster *et al.*, 2010; Ohsako *et al.*, 2010; Rebourcet *et al.*, 2010). Some studies have suggested that exposure to dioxin-like PCBs or PCB mixtures *in utero* or during breastfeeding is associated with reduced testosterone levels in male rat offspring, but the timing of the low testosterone levels varies in different studies (Faqi *et al.*, 1998; Kim *et al.*, 2001; Yamamoto *et al.*, 2005). In a study on pubertal boys belonging to Faroese birth cohort, boys having high testosterone concentrations at 14 years of age tended to have high PCB levels (sum of PCBs 138, 153, and 180) in umbilical cords (Mol *et al.*, 2002). We found no significant associations between PCDD/F WHO-TEq levels (i.e. 2,3,7,8-TCDD equivalent quantities) in placenta and reproductive hormone levels at 3 months of age. In the Finnish material, placenta PCB WHO-TEq levels associated positively with infant LH levels at the age of 3 months. This could theoretically suggest an association between PCB-exposure and some form of Leydig cell dysfunction. However, this association was not found in the Danish material, and it may also represent a chance finding.

The PCDD/F WHO-TEq level, PCB WHO-TEq level (only after adjustment for confounding factors), total-TEq level and the sum of PCBs was significantly higher in Danish control placentas than in Finnish. We have previously found similar country differences in levels of some organochlorine pesticides in placenta and breast milk (Shen *et al.*, 2008; Krysiak-Baltyn *et al.*, 2010) and in breast milk levels of some dioxin and PCB congeners (Krysiak-Baltyn *et al.*, 2010). No outstanding differences in regulation of the chemicals between Finland and Denmark were observed (Krysiak-Baltyn *et al.*, 2010). However, dietary intakes of dioxins are from different sources in Denmark and Finland. In Denmark, meat and milk have been the most important sources (King, 1999). In Finland, agricultural products have been quite clean, and most intake is from Baltic fish, especially Baltic herring (Kiviranta, 2005). Much of meat and milk dioxins can be explained by airborn fallout to fields and pastures, Baltic Sea contamination is both airborn and from industrial wastewater emissions (Koistinen *et al.*, 2008). When comparing our results to a previous Finnish study evaluating placenta levels of 17 dioxins and 36 PCBs in 1995–1999 (Laisi *et al.*, 2008), a decreasing tendency of dioxin and PCB placenta levels in Finland was observed. This is in line with observations concerning decreasing dioxin breast milk levels in several countries (van Leeuwen & Malisch, 2002). In addition, the Danish placenta levels of dioxins and PCBs seem to be lower than the levels described in the previous Finnish study (Laisi *et al.*, 2008). Median Finnish placenta levels were slightly lower and median Danish levels slightly higher (except for PCBs) than the levels reported in a study representing general Taiwanese population at the turn of the century: Median PCDD/F, PCB and total WHO-TEq levels in placenta were 10.2 pg/g fat, 2.7 pg/g fat and 12.8 pg/g fat, respectively, in the Taiwanese material (Chao *et al.*, 2007). Both the Finnish and Danish dioxin levels seem to be lower than the placenta dioxin levels reported in a Vietnamese material (range 14.6–48.5 pg WHO-TEq/g fat) (Feshin *et al.*, 2008).

Our negative findings suggest that at current exposure levels in Finland and Denmark, dioxins and PCBs are not the main chemicals that contribute to the aetiology of congenital cryptorchidism. This does not exclude the possibility that they are a part of the overall chemical load that can have adverse effects. The search for specific chemical contaminants that may contribute significantly to the pathogenesis of cryptorchidism must continue.

## Conclusions

No associations between placenta levels of dioxins or PCBs and congenital cryptorchidism were found in this study. Significant differences between countries in chemical levels were observed, corroborating previously described differences between Finland and Denmark in the levels of persistent chemicals.
